# Perspectives of Patients and Professionals on Information and Education After Myocardial Infarction With Insight for Mixed Reality Implementation: Cross-Sectional Interview Study

**DOI:** 10.2196/17147

**Published:** 2020-06-23

**Authors:** Alexander D Hilt, Kevin Mamaqi Kapllani, Beerend P Hierck, Anne C Kemp, Armagan Albayrak, Marijke Melles, Martin J Schalij, Roderick W C Scherptong

**Affiliations:** 1 Department of Cardiology Leiden University Medical Center Leiden Netherlands; 2 Faculty of Industrial Design Engineering Delft University of Technology Delft Netherlands; 3 Department of Anatomy and Embryology Leiden University Medical Center Leiden Netherlands

**Keywords:** human factors, myocardial infarction, mixed reality, patient education, patient experience, PROM

## Abstract

**Background:**

Patient education is crucial in the secondary prevention of cardiovascular disease. Novel technologies such as augmented reality or mixed reality expand the possibilities for providing visual support in this process. Mixed reality creates interactive digital three-dimensional (3D) projections overlaying virtual objects on the real-world environment. While augmented reality only overlays objects, mixed reality not just overlays but anchors virtual objects to the real world. However, research on this technology in the patient domain is scarce.

**Objective:**

The aim of this study was to understand how patients perceive information provided after myocardial infarction and examine if mixed reality can be supportive in this process.

**Methods:**

In total, 12 patients that experienced myocardial infarction and 6 health care professionals were enrolled in the study. Clinical, demographic, and qualitative data were obtained through semistructured interviews, with a main focus on patient experiences within the hospital and the knowledge they gained about their disease. These data were then used to map a susceptible timeframe to identify how mixed reality can contribute to patient information and education.

**Results:**

Knowledge transfer after myocardial infarction was perceived by patients as too extensive, not personal, and inconsistent. Notably, knowledge on anatomy and medication was minimal and was not recognized as crucial by patients, whereas professionals stated the opposite. Patient journey analysis indicated the following four critical phases of knowledge transfer: at hospital discharge, at the first outpatient visit, during rehabilitation, and during all follow-up outpatient visits. Important patient goals were understanding the event in relation to daily life and its implications on resuming daily life. During follow-up, understanding physical limitations and coping with the condition and medication side effects in daily life emerged as the most important patient goals. The professionals’ goals were to improve recovery, enhance medication adherence, and offer coping support.

**Conclusions:**

There is a remarkable difference between patients’ and professionals’ goals regarding information and education after myocardial infarction. Mixed reality may be a practical tool to unite perspectives of patients and professionals on the disease in a more even manner, and thus optimize knowledge transfer after myocardial infarction. Improving medication knowledge seems to be a feasible target for mixed reality. However, further research is needed to create durable methods for education on medication through mixed reality interventions.

## Introduction

Coronary artery disease is a major cause of mortality in developed countries, leading to roughly 1.5 million deaths annually worldwide [1,2]. Improvements in early recognition of the disease and treatment have significantly decreased the mortality rate after myocardial infarction over the last few decades [3]. However, increased complexity in treatment and long-term care makes educating patients about their disease a challenge for health care professionals. Guiding patients through complex terminology, pathophysiological concepts, and extensive treatment options in a limited time frame is a stressful and demanding process for both health care professionals and patients [4].

Improvements have been made regarding patient information and education through extensive written information, informational videos, or digitalized “how does it look” visual models [5-8]. Attempts at improving education in patients following myocardial infarction are scarce and have mainly focused on care processes and anatomical knowledge [9-11]. With rapid development of new technologies such as virtual reality [12] or more recent mixed reality modalities [3], patient information and education approaches have also been changing [13-15]. Mixed reality creates interactive digital three-dimensional (3D) projections that are viewed through a head-mounted display such as Microsoft HoloLens.

With the introduction of this new technology, the possibilities to support daily care increase, in particular regarding improvements in anatomical knowledge. However, this adds another layer of complexity to the care process. The question therefore remains as to how to best establish the added value of implementing a new technology such as mixed reality in the educational process on a patient level.

To optimize the process of patient information and education after myocardial infarction, information should add to the sustainability of health and disease prevention [16]. The latter aspect is a particular cornerstone of myocardial infarction care [1]. Toward this end, the aim of this study was to assess how patients perceive patient information and education resources offered after myocardial infarction without the use of a mixed reality app. A secondary aim was to identify targets for mixed reality within the domain of patient information and education after myocardial infarction.

## Methods

### Design

This was a cross-sectional interview study. Ethical approval for the project was obtained through the local medical ethics committee of Leiden University Medical Center (protocol number P18.132).

### Study Population

Twelve consecutive patients who visited the dedicated outpatient clinic for patients after myocardial infarction were asked to participate in the study. The patients were at various stages in their recovery, ranging between 1 and 12 months after the initial myocardial infarction. In addition, two cardiologists, two nurse specialists, one psychologist, and one sexologist were included in the study to obtain the professional stakeholders’ point of view. Demographic data such as age, gender, occupation, and time of interviewing (1, 3, 6, or 12 months after myocardial infarction) were collected. Additionally, clinical demographics such as comorbidities (smoking, hypertension, diabetes mellitus), initial diagnosis (ST-elevation myocardial infarction [STEMI] or nonST-elevation myocardial infarction [NSTEMI]), culprit lesion of the myocardial infarction, maximum troponin levels at admission, and left ventricular ejection fraction (LVEF%) at hospital discharge were collected from the electronic medical record.

### Semistructured Interviews and Questionnaires

In line with existing value-based health care literature, generic Patient Reported Outcome Measure tools were used in the current study [17]. First, we evaluated whether patients felt that the information provided during clinical care was sufficient, if they understood what medications they were taking, and the purpose of the medication. Second, we assessed the extent of knowledge the patients had about their disease and the effect on cardiac function.

We conducted semistructured interviews to assess patients’ knowledge about personal myocardial infarction characteristics. A list of questions (Multimedia Appendix 1) was used to conduct the interviews. The first part of the interview included questions related to social and demographic factors. The second part of the interview consisted of questions related to myocardial infarction-specific knowledge. The last part of the interview included the Generic Short Patient Experiences Questionnaire (GS-PEQ). This questionnaire was originally developed to be used in multiple health care settings to evaluate the patient experience through standardized questions in addition to other qualitative measures such as semistructured interviews [18]. According to the aim of this study, the GS-PEQ was used to gain insight into patients’ opinions about their experience during clinical care.

Since one of the core features of mixed reality is visualizing complex 3D models to interact with, it is relevant to understand if patients have a basic understanding of cardiac anatomy. Therefore, the level of knowledge about coronary artery disease was tested. Two forms were used: one that showed a representation of the coronary arteries, in which the patients could label the vessels that were occluded/obstructed in their case (Multimedia Appendix 2), and the other included two diagrams representing the simplified cardiac anatomy of the heart on which patients could label the area affected and how it is related with pump function, if applicable (Multimedia Appendix 3). All interviews were audio-recorded and subsequently transcribed.

### Semistructured Interview With Professionals

To gain insights into the process and map the professionals’ perspective on information provision during the patient journey, semistructured interviews were conducted with professionals engaged in the treatment of patients with myocardial infarction. A list of questions was used to guide the interview (Multimedia Appendix 4), which were adapted according to the specific professional activities. The main focus of the interviews was to identify the materials professionals use to interact with patients, the dynamics of the consultations they conduct, and how and when they consider the need to educate patients.

### Analysis

#### Content Analysis

Content analysis was used to structure all of the qualitative data from the interviews, which were summarized through descriptive statistics and examples of general comments. Numerical data are presented as means (SD) and categorical data are presented as proportions. GS-PEQ outcomes were used to structure the patient journey (see further description below); these outcomes were then used for the establishment of themes relevant to both professionals and patients.

#### Patient Journey Analysis

A patient experience journey was created via a standardized approach to analyze the patient experience within the dedicated care track of myocardial infarction treatment, with specific attention paid to knowledge transfer between professionals and patients [19]. For this purpose, the patients underwent observations during outpatient visits at our department, and were then interviewed subsequently with the researchers and were asked to fill out questionnaires consecutively.

Patient journey mapping is a frequently used method among design engineers, but is relatively new in the medical domain. This approach combines several methods to best understand the patient’s experience by dividing the management of a specific condition, or process such as education, into a series of consecutive steps or events [19]. The mapping is performed using data collected from semistructured interviews, questionnaires, and observations. Combining these data, the result of the final patient journey offers a description of the dedicated care track as seen by professionals and experienced by the patient. In this study, the patient journey analysis included descriptions of the main event (myocardial infarction), acute treatment and total duration of treatment, the environment in which treatment takes place, and interactions with professionals. Importantly, this analysis can highlight the key points of knowledge transfer, materials of interaction, patient concerns, patient goals, professional goals, and guide eventually possible mixed reality interventions throughout the patient experience when treated for myocardial infarction.

## Results

### Demographics of the Study Population

A total of 12 patients and 6 professionals were interviewed in this study. There were 9/12 (75%) and 3/6 (50%) men in the patient and professional group, respectively. The average age of the patients and health care professionals was 62.7 (SD 10.4) years and 43.2 (SD 9.6) years, respectively. Among the patients, there were 2/12 (17%) current smokers, and the remaining 10 (83%) had stopped smoking after myocardial infarction. Six (50%) patients suffered from hypertension and 2/12 (16%) had diabetes. The majority of patients (10/12, 83%) suffered from a STEMI, with a common culprit vessel being the left anterior descending artery (6 patients, 50%, Table 1). The average LVEF at discharge after myocardial infarction was 49.8% (SD 6.8%) and the average maximum troponin release was 8140.3 ng/L (SD 13.623).

### General Experience

Six (50%) patients (all men) indicated that the information shared (written or spoken, presented in analog or digital format) was too extensive and repetitive, whereas one male patient stated that more information was needed. Overall, the patients indicated that clinicians were able to provide them with sufficient care, specifically regarding information on their diagnosis. However, 9/12 (75%; 2 women, 7 men) patients noted that they were not involved in specific decisions regarding their treatment process. Only one male patient reported that the given treatment was incorrect according to his own judgment (Table 2).

From the professionals’ perspective, optimal timing for information exchange is perceived at the first visit at 1 month after myocardial infarction (6/6, 100%). All professionals (6/6, 100%) also stated that they wish to educate patients in a understandable and complete manner, although the timeframe is perceived to be too short in the outpatient setting.

**Table 1:** Demographic overview of the patients.

Sex	Age (years)	Profession	Interview time after MI^a^	Smoking	HT^b^	DB^c^	LVEF (%)^d^	Tmax^e^ (ng/L)	Type of MI	Culprit vessel
Female	61	Administrative assistant	1 month	Stopped	Yes	Yes	39	10,553	STEMI^f^	LAD^g^
Male	62	Lawyer	1 month	Stopped	No	No	58	50,000	STEMI	LAD
Male	74	Vice principal	3 months	Stopped	No	No	58	1160	STEMI	RCA^h^
Male	52	Manager	3 months	Stopped	Yes	No	58	1504	NSTEMI^i^	RCA
Male	57	Foreman	6 months	Yes	Yes	No	48	8389	STEMI	LAD
Female	63	Nurse	6 months	Stopped	Yes	No	58	20	STEMI	RCA
Male	54	Engineer	6 months	Stopped	No	No	44	5659	STEMI	LAD
Male	56	Information technology consultant	6 months	Stopped	Yes	No	50	5308	STEMI	RCA
Male	64	Dentist	12 months	Yes	No	Yes	49	3990	STEMI	LAD
Male	78	Architect	12 months	Stopped	No	No	45	2078	NSTEMI	D1^j^
Male	82	Truck driver	12 months	Stopped	No	No	48	8406	STEMI	RCx^k^
Female	49	Housewife	12 months	Stopped	Yes	No	42	622	STEMI	LAD

^a^MI: myocardial infarction.

^b^HT: hypertension.

^c^DB: diabetes mellitus.

^d^LVEF: left ventricular function at infarction.

^e^Tmax: maximal troponin release.

^f^STEMI: ST-elevation myocardial infarction.

^g^LAD: left anterior descending artery.

^h^RCA: right coronary artery.

^i^NSTEMI: nonST-elevation myocardial infarction

^j^D1: diagonal branch.

^k^RCx: circumflex artery.

**Table 2:** Generic Short Patient Experiences Questionnaire (GS-PEQ) (N=12).

Question	Agree, n (%)
Did the clinician talk to you in a way that was easy to understand?	12 (100)
Do you have confidence in the clinicians’ professional skill?	9 (75)
Did you get sufficient information about your diagnosis?	11 (92)
Did you perceive the treatment as adapted to your situation?	11 (92)
Were you involved in decisions regarding your treatment?	3 (25)
Did you perceive the institution’s work to be well organized?	12 (100)
Did you have to wait before you were admitted for services at the institution?	12 (100)
Overall, was the help and treatment you received at the institution satisfactory?	11 (92)
Did you benefit from the care given at the institution?	11 (92)
Do you believe that you were in any way given incorrect treatment?	1 (8)

### Medication Usage

Six of the 12 (50%; 2 women, 4 men) patients were unaware of the type of medication they were taking and its purpose. In addition, 10/12 (83%; 3 women, 7 men) patients considered the medication to influence their recovery in a negative manner.

From the professionals’ perspective, written and hand-drawn educational information were stated as the most frequently used materials for both providing medication information and anatomical knowledge transfer (6/6, 100%), followed by video (3/6, 50%) and Microsoft PowerPoint presentations (1/6, 17%). Accurate insight on medication (“what medication do you use and why?”) among patients was perceived to be poor by professionals; 4/6 (85%) of the professionals stated that they frequently encounter this problem in the outpatient setting. The professionals equally stated a desire to educate patients on the cardioprotective function as completely as possible (6/6, 100%).

### Anatomical Knowledge

**Figure 1:**
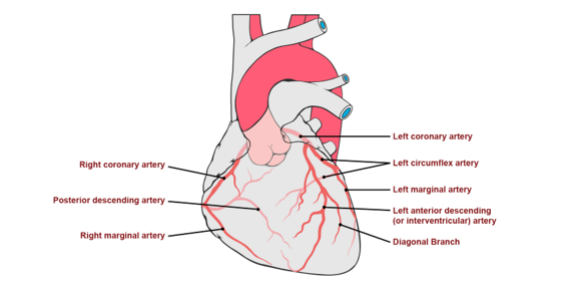
Representation of the coronary arteries. Patients were asked the following: “Could you please tick on the boxes which of your arteries have been affected, if any? Also, on the left illustration, draw the parts affected after the myocardial infarction.”

**Figure 2:**
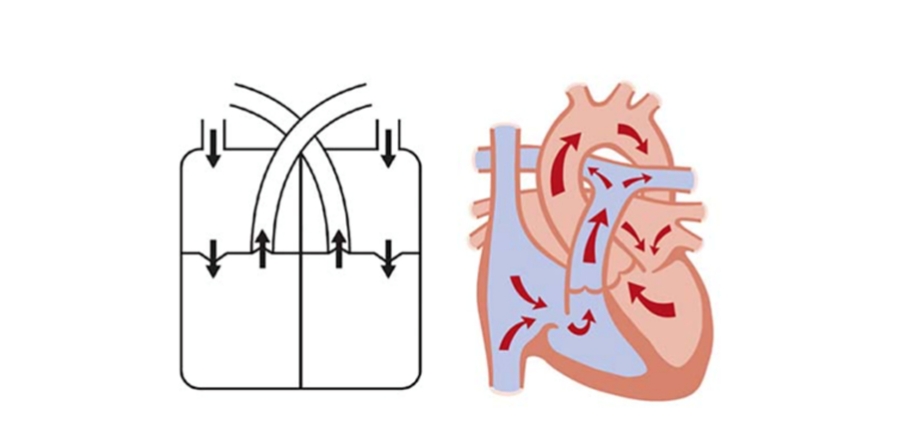
Representation of heart blood circulation (left) and the main parts of the heart (right). Patients were asked: “Could you please tick on the boxes corresponding to the parts of your heart that have been affected, if any? Also, draw the affected parts on the left illustration.”

Regarding anatomical knowledge, 4/12 (33%; 1 woman, 3 men) patients were aware of the culprit vessel (Figure 1, Table 3) and 4/12 (33%; 1 woman, 3 men) knew the affected site (Figure 2, Table 4). Only 2/12 (17%, both men) patients knew the area of the heart that was affected by the culprit lesion: 10/12 (83%; 3 women, 7 men) patients had no knowledge of the relationship between the diseased (culprit) vessel and the effect on their heart. Six (50%; 1 woman, 5 men) of the patients noted that this type of information was not relevant to them. Examples of comments given by patients are shown in Textbox 1.

All professionals (6/6, 100%) stated that there should be more time available to educate patients on an anatomical understanding of myocardial infarction.

**Table 3:** Culprit lesion knowledge (also see Figure 1).

Patient	Culprit lesion	Correctly shown in figure?
1	LAD^a^	No
2	LAD	Yes
3	RCA^b^	No
4	RCA	No
5	LAD	No
6	RCA	No
7	LAD	Yes
8	RCA	Yes
9	LAD	No
10	D1^c^	Yes
11	RCx^d^	No
12	LAD	No

^a^LAD: left anterior descending artery.

^b^RCA: right coronary artery.

^c^D1: left anterior descending artery diagonal branch.

^d^RCx: circumflex artery.

**Table 4:** Affected site knowledge (see Figure 2).

Patient	Correct site shown
1	No
2	No
3	No
4	No
5	Yes
6	No
7	Yes
8	Yes
9	No
10	No
11	No
12	Yes

**Example patient comments related to information exchange with professionals.**

- Overall information exchange

“Too much information to comprehend at once”

“I really don’t need to know all what they tell me”

“I really wanted to know way more than they tell me”

- Medication-related information

“I have no idea what I am taking”

“I am in charge over my body and I want to live a great life without medication”

“So many pills! That is a big problem for me, but what can I do?”

“I have different kind of colors and sizes, don’t know what they do”

### Patient Experience Journey: Care Track and Opportunities for Mixed Reality

#### Mixed Reality Information Exchange Goals

**Figure 3:**
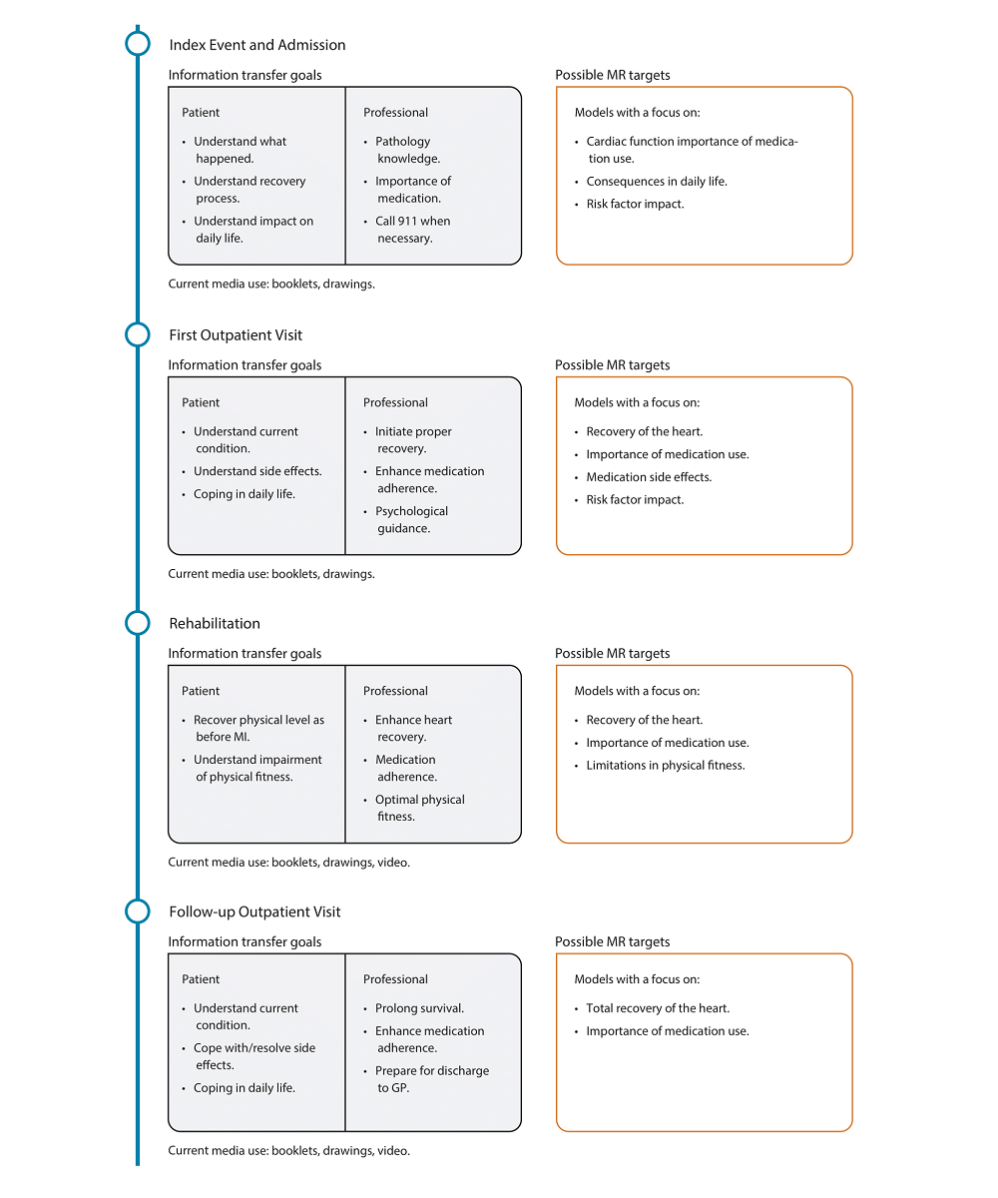
Overview of patient experience regarding knowledge transfer and mixed reality (MR) possibilities.

Figure 3 shows the key elements regarding knowledge transfer after myocardial infarction, and Multimedia Appendix 5 provides a full overview of the patient journey. The patient journey includes the goals of both patients and professionals at each step of the care track. Key points regarding information transfer were assessed at hospital discharge, during the first outpatient visit, and during the rehabilitation initiation. Information exchange during these phases is currently performed using drawings, the postmyocardial infarction care track information booklet, and videos (Figure 3).

#### Discharge and First Outpatient Visit

Patient goals at discharge were understanding what happened, what the current condition is, and how it affects their daily life. Professionals focus on describing the event, relating it to risk factors, and stressing the importance of seeking attention when similar symptoms that may indicate a myocardial infarction are experienced.

Goals at the first outpatient visit were the same as those at discharge with the addition of understanding the side effects of medications as well as coping with the disease in daily life. Professionals focus on optimal recovery through optimal medication adherence, stressing the importance of rehabilitation and providing psychological guidance when needed. Mixed reality can help to visually support the patient’s clinical state when they leave the hospital, as well as stressing the importance of medication, risk factor impacts such as smoking, and possible side effects of medication that are to be expected (Figure 3).

#### Rehabilitation and Outpatient Follow-Up

During rehabilitation, patient goals focus on physical fitness in terms of understanding the impairment of the disease and reaching the premyocardial infarction level of fitness. Professionals focus on increasing physical fitness through exercise and support recovery by stressing medication adherence.

During outpatient follow-up, patient goals focus on adjusting to the current health condition in daily life and understanding the potential side effects that may occur. Professionals focus mainly on prolonging survival by optimizing medication adherence and lifestyle as well to prepare patients for eventual discharge to the family physician. Mixed reality can visually support the physical condition of patients by showing the current state of heart function and its effect on physical fitness, along with the state of recovery of the heart and highlighting the long-term importance of medication on survival.

## Discussion

### Principal Findings

Overall, the results of the current study demonstrate that patients and clinical staff have very different opinions about the overall information shared during outpatient clinical visits, anatomical knowledge, and medication. First, patients reported that the information shared was too extensive and superfluous, whereas staff members stated a desire to share more information. Second, patients perceived medication as a hurdle toward their recovery, whereas professionals viewed the medication as an important part of their recovery. Third, the anatomical knowledge of patients was minimal regarding the culprit lesion and its effect on cardiac function. The patient journey in this regard showed that patients transition from a state of uncertainty to a state of confidence; however, the lack of knowledge remains and reassurance by health care providers is regarded as important.

### Patient Information Education After Myocardial Infarction

Throughout the year following myocardial infarction, patients see roughly 4 clinical specialists and often also see a psychologist or sexologist, all of whom elaborate on the same concept of myocardial infarction. However, our outcomes suggest that patient knowledge of simple anatomical and physiological concepts of heart disease remains minimal. Furthermore, patients regard medication as a hurdle toward recovery although it is the hallmark of secondary prevention in cardiovascular care.

Scott et al [20] found that patients ranked explanation of anatomical and pathophysiological concepts as well as medication information at high importance after myocardial infarction; however, the effect of teaching these aspects to patients regarding their long-term survival is not known. It is also questionable if teaching of these concepts is essential to reach the goal of preventing new myocardial infarction, and evidence in this regard is lacking.

Our patients received identical information after myocardial infarction; however, they seem to have gained little understanding from this education, and mainly perceived the information provided as too extensive, which was not considered to be in line with their own goals. Therefore, our study highlights room for improvement in patient information education after myocardial infarction.

Professional goals (prevention of new myocardial infarction) and patient goals (living a normal life) differ to a striking degree (Figure 3). Although the necessity of teaching anatomical and pharmacological concepts might be debatable, patient care regarding information exchange should be in line with the goals of patients to support patient-centered care [17]. To unite these goals, the interaction between a patient and professional needs to be assessed and reevaluated based on the results of our study. When this information exchange is goal-oriented, learning and adoption of new information will be more effective, as stated by the cognitive load theory proposed by Sweller [21]. This theory states that the methods of information exchange should promote a low extraneous cognitive load (ie, presentation of information). Conventional methods (ie, booklets) create high levels of extraneous load, whereas visual methods create a low extraneous load [21]. Therefore, use of a mixed reality app might effectively aid in generating a low extraneous load and offer a new method of learning. This warrants further research, particularly if implementation of mixed reality for patient information education can lead to improvement of medication adherence.

### Identifying Targets for Mixed Reality

As seen in the patient journey analysis, there are certain points at which mixed reality may provide solutions in patient information exchange. Certain targets might provide less information, but will nonetheless be aligned with actual patient data, including guidance on the effect of medication on their current health condition.

Mixed reality has been recently popularized by the development of Google Glass and subsequently Microsoft HoloLens, released in March 2016 [14]. HoloLens can project interactive 3D images in the field of vision of the user and recognize the environment owing to the presence of four environment-sensing cameras, a depth camera, and a light sensor. Apart from recognizing the environment, HoloLens also memorizes it, thereby reducing the time required for the next interaction. HoloLens can also recognize human gestures to enable interaction and teamwork around the same projected objects owing to integration of human understanding software such as spatial sound, gaze tracking, gesture input, and voice support [22].

Table 5 provides an overview of the different types of media available for mixed reality and their usability, along with a summary of usability and capabilities. The main capabilities of HoloLens to be considered in the outpatient setting are: (i) recognize and interact with the environment, to choose the best environment for the interventions and base the design accordingly; (ii) project 3D images that can rotate, scale, or move; and (iii) encourage teamwork by enabling doctors and patients to collaborate through synchronization of doctor and patient images in space, giving them an opportunity to collaboratively study the model. Through these capabilities, mixed reality creates new ways of collaboration between the patient and professional. Recent studies have tested mixed reality for medical training [15] and as a surgical assistive technology [23]. For medical students, especially those with lower visual-spatial abilities, mixed reality was shown to significantly improve 3D knowledge acquisition [24]. However, no apps currently exist that use mixed reality specifically to educate myocardial infarction patients or to improve their experience during the treatment after myocardial infarction.

Our results indicate that mixed reality may be of aid in compiling patient-specific data in one model such as a simplified model of the heart and coronary anatomy using radiographic and ultrasound data. This may be used at the end of the hospital stay when patients are fit to go home, and when uncertainties are present. A mixed reality intervention at discharge can provide a crude overview of myocardial infarction and the importance of medication and education on minimizing risk factors such as smoking. This technology can be used consecutively throughout all outpatient visits, compiling cardiac function in the model and thereby offering the possibility to use one model consecutively. Furthermore, mixed reality can be used to explain the effects of medication on long-term survival.

**Table 5:** Media types and usefulness in patient education.

Usability and capability	Mixed Reality (HoloLens)	Augmented Reality	Virtual Reality	Video	Text and Images
Interaction between two or more users	Full	Partial	Partial	No	No
Movement	Yes	Yes	Yes	Partial	No
Environment aware	Yes	Partial	No	No	No
Device needed	Yes	Yes (phone)	Yes (phone)	Yes (TV, computer, or phone)	No

### Medication as a Specific Target for Mixed Reality

The patients included in our study perceived medication as a hurdle toward recovery. They indicated that this is mainly coupled to side effects but also that the beneficial effects are unclear (despite all information provided). Optimal medical therapy after myocardial infarction is the cornerstone of cardioprotective care and is essential in preventing new events [12]. This has been stressed by both the European and American cardiology societies [25]. However, nonadherence to medication is a common problem [26]. Through the years, attempts have been made to improve medication nonadherence; however, it remains a challenge to create sustainable interventions [27].

The patient journey analysis suggested that reassurance is important for patients to understand their condition such as whether or not they are physically fit. Clear explanation of medication benefits on their health and daily life may resolve the lack of understanding of medication effects and potentially lower the need for reassurance.

Tailoring education to patient-specific features and needs such as medication adherence seems to be effective, which has been proposed in other studies. Nieuwkerk et al [28] demonstrated that by clarifying the effect and importance of statins visually, low-density lipoprotein cholesterol levels can be reduced along with an increase in the intake of statins. A randomized study conducted by Jones et al [29] in 2015 showed that providing visual education after myocardial infarction improved illness and medication perceptions in the intervention group. A similar approach may be feasible in patients after myocardial infarction that are offered a new form of education through mixed reality. A model could be developed, not focusing on anatomy per se but rather on statin use and the effect on the patient’s cardiovascular health, such as by demonstrating atherosclerosis in coronary vessels, which is targeted by statin therapy [1]. The effect of such a mixed reality intervention could be measured according to assessing medication beliefs and illness perceptions.

Further research is needed to test our assumptions. Importantly, the implementation and evaluation of a mixed reality app in the elderly should be undertaken. Along with an aging population, potential users will be between 60 and 80 years old, which is accompanied by different forms of disabilities (ie, impaired vision, hearing, or cognitive function) that can complicate use. However, mixed reality seems to be an accessible and feasible tool in the elderly, as highlighted by Rohrbach et al [30] in patients with Alzheimer disease. Since patients with Alzheimer disease comprise a complex patient group, it is feasible to assume that patients with no cognitive impairments might also benefit from mixed reality apps.

In this era of rapidly evolving technology that brings new opportunities regarding patient information education, it is important to thoroughly evaluate how these technologies can be used in a changing medical setting and with what goal in mind, especially given the sparsity of research on the topic.

### Limitations

There are certain limitations to our study. First, all interviews were conducted in a group of patients and professionals belonging to a single hospital. Using a different group of professionals and patients from different hospitals and social backgrounds, different outcomes may be generated concerning patient information education. Second, and following this point, the small study size could have led to overestimating the assumptions such as the problems patients have with medication. Further investigation on this subject is therefore warranted. Third, observational interview studies have inherent biases (such as responder bias or social desirability bias). This can also be corrected using a larger-scale study.

### Conclusion

We identified a remarkable difference between the goals of patients and health care professionals regarding information and education after myocardial infarction. Mixed reality may be a practical tool to unite the perspectives of patients and professionals on the disease in a more even manner, and thus optimize knowledge transfer after myocardial infarction. Medication understanding seems to be a feasible target for mixed reality. However, further research is needed to develop durable methods for education on medication through mixed reality.

## Appendix

Multimedia Appendix 1 Patient interviews at the outpatient clinic visit (translated in English). The following questions were used to obtain information about the patient, the crucial points through the journey, and their anatomy knowledge.

Multimedia Appendix 2 Images used during interviews: coronary anatomy.

Multimedia Appendix 3 Images used during interviews: understanding heart anatomy and function.

Multimedia Appendix 4 Questions used in clinical staff interviews.

Multimedia Appendix 5 Full patient journey (added as a vector file).

## Abbreviations

3D: three-dimensional
GS-PEQ: Generic Short Patient Experiences Questionnaire
LVEF%: left ventricular ejection fraction
NSTEMI: nonST-elevation myocardial infarction
STEMI: ST-elevation myocardial infarction
