# Supplementary material for: Perspectives of Patients and Professionals on Information and Education After Myocardial Infarction With Insight for Mixed Reality Implementation: Cross-Sectional Interview Study
Source: JMIR Hum Factors. 2020 Jun 23;7(2):e17147. doi: 10.2196/17147 (PMC7381062; doi:10.2196/17147)
Supplement: Multimedia Appendix 1 [file humanfactors_v7i2e17147_app1.docx]

**Appendix A: patient interviews at the outpatient clinic visit (translated in English).**
The following questions are used to obtain information about the patient, the crucial points through the journey and their anatomy knowledge:

| About the patient profile | |
| --- | --- |
| 1 | What is your age? |
| 2 | What is your profession? (follow up) Did you start with work after the event? Are there any complications? |
| 3 | Are you a smoker? Have you smoked in the past? |
| 4 | What is your marital status? – and do you live alone, with family, someone to support (give some options). |
| 5 | What outpatient visit is this? (first, second, third or fourth). |
| 6 | Did you have consultations with the cardiology departments psychologist? |
| 7 | Are you going to the rehabilitation center? |
| 8 | Which one of the previous services do you find more helpful? Why? |
| 9 | Have you been prescribed any medication? Which one? |
| 10 | Do you understand what are the effects of the medication on your body? Can you elaborate (do you feel any consequence)? |
| About the patients pathology | |
| 1 | What is the name of your disease? |
| 2 | Who diagnosed it? |
| 3 | What treatment did you receive? - Were you already receiving treatment/medication before the event? |
| 4 | What are the consequences of your disease? |
| 5 | How are you informed about your pathology? - Doctors, Internet, Friends… - If a combination, which source did you like the most? |
| 6 | What materials did you receive at the outpatient clinic? - Text, drawings, videos. - Which one did you like the most? |
| 7 | Could you please fill-in the next drawings: (see Appendix B and C) |
| Evaluation questions (GS-PEQ) | |
| 1 | Did the clinicians a talk to you in a way that was easy to understand? |
| 2 | Do you have confidence in the clinicians' a professional skills? |
| 3 | Did you get sufficient information about your diagnosis/afflictions? |
| 4 | Did you perceive the treatment as adapted to your situation? |
| 5 | Were you involved in decisions regarding your treatment? |
| 6 | Did you perceive the institution's work as well organised? |
| 7 | Did you have to wait before you were admitted for services at the institution? |
| 8 | Overall, was the help and treatment you received at the institution satisfactory? |
| 9 | Overall, what benefit have you had from the care at the institution? |
| 10 | Do you believe that you were in any way given incorrect treatment (according to your own judgment)? |
| Extra | |
| 1 | Did you forget to ask any particular question after your outpatient visit? |
