# Supplementary material for: Perspectives of Patients and Professionals on Information and Education After Myocardial Infarction With Insight for Mixed Reality Implementation: Cross-Sectional Interview Study
Source: JMIR Hum Factors. 2020 Jun 23;7(2):e17147. doi: 10.2196/17147 (PMC7381062; doi:10.2196/17147)
Supplement: Multimedia Appendix 2 [file humanfactors_v7i2e17147_app2.docx]

*
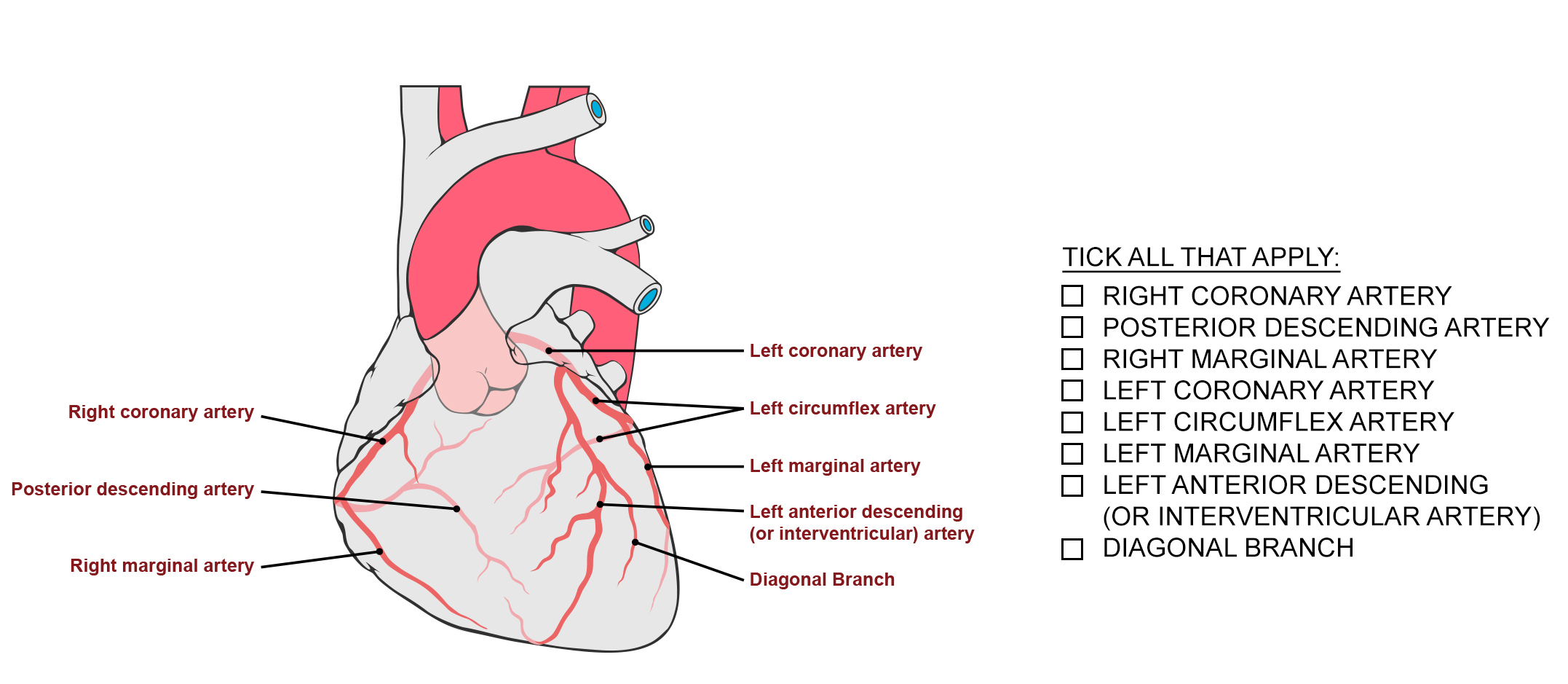
***Appendix B: Images used during interviews: coronary anatomy.**

*The following diagram is a representation of the coronary arteries. Patients were asked the following: ‘Could you please tick on the boxes which of your arteries have been affected, if any? Also, draw on the left illustration which parts are affected after the myocardial infarction’.*
