# Supplementary material for: Perspectives of Patients and Professionals on Information and Education After Myocardial Infarction With Insight for Mixed Reality Implementation: Cross-Sectional Interview Study
Source: JMIR Hum Factors. 2020 Jun 23;7(2):e17147. doi: 10.2196/17147 (PMC7381062; doi:10.2196/17147)
Supplement: Multimedia Appendix 3 [file humanfactors_v7i2e17147_app3.docx]

**Appendix C. Images used during interviews: understanding heart anatomy and function.**


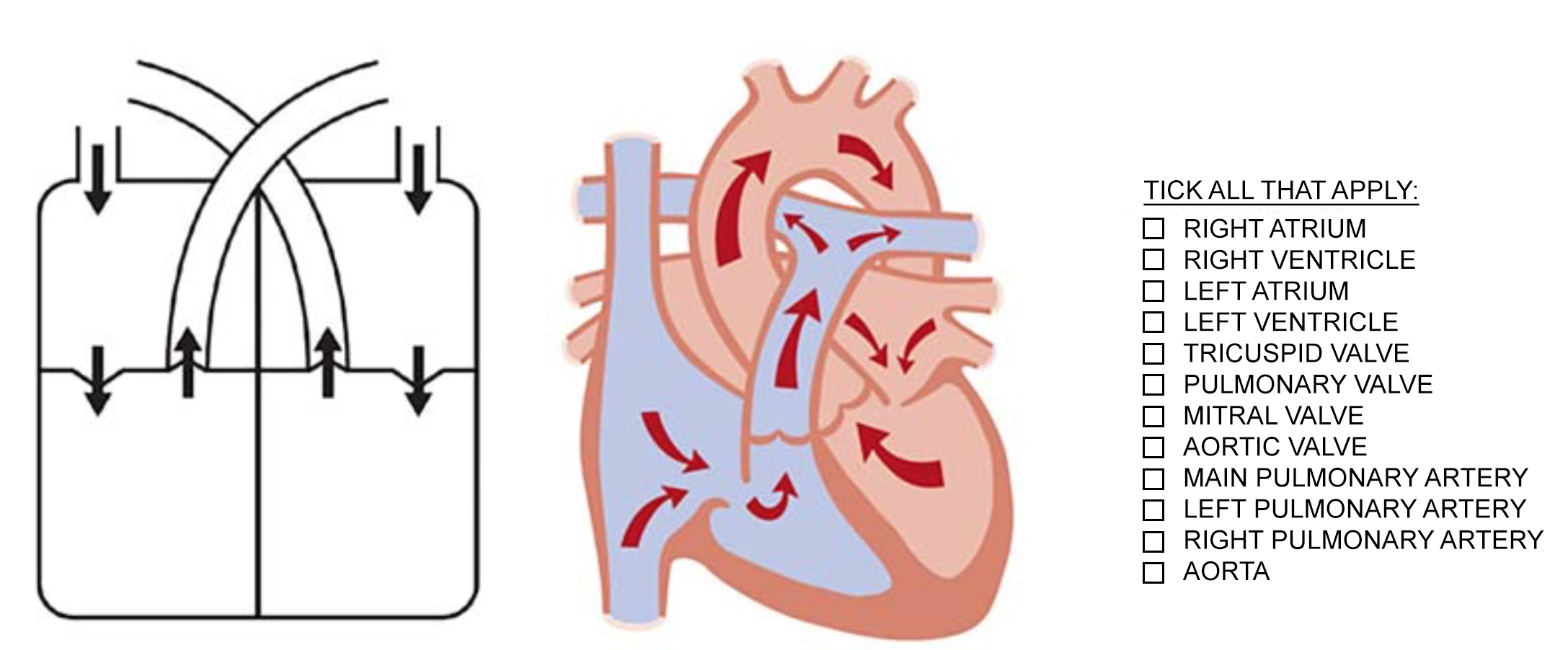
The following diagrams are a representation of the heart blood circulation and cardiac anatomy. The left one is a simplification of the blood flow, the right one is an illustration of the section of the heart. Patients were asked to*: ‘Could you please tick on the boxes which parts of your heart have been affected, if any? Also, draw on the left illustration were affected’.*
