# Supplementary material for: Perspectives of Patients and Professionals on Information and Education After Myocardial Infarction With Insight for Mixed Reality Implementation: Cross-Sectional Interview Study
Source: JMIR Hum Factors. 2020 Jun 23;7(2):e17147. doi: 10.2196/17147 (PMC7381062; doi:10.2196/17147)
Supplement: Multimedia Appendix 4 [file humanfactors_v7i2e17147_app4.docx]

**Appendix D: Questions used in clinical staff interviews.**
The following questions are used to obtain information about the clinical staff and their interaction with the patients:

| About the nurse | |
| --- | --- |
| 1 | What is your age? |
| 2 | What is your specialization? How many years of experience do you have? |
| 3 | How many patients do you interview per week? |
| About the interview dynamics | |
| 1 | How long are your interviews with patients? |
| 2 | What steps does the protocol include in each interview? (long answer, elaborate on steps and keywords (sexologoy, medication, lifestyle, work, …)). |
| 3 | How would you define your role during the interview? (diagnosis, gather information, patient guidance, a mixture of the previous). |
| 4 | Do you use any materials such as texts, images or videos during the interview? (if so, related to what?) |
| 5 | What are the main differences between the four interviews during the outpatient clinic visit? |
| 6 | What are the main concerns of the patients in general, and on each one? |
| 9 | Do patients talk in a way that was easy to understand? |
| 10 | Do you get sufficient information from the patient? |
| 11 | Do you think that the treatment is generic or rather customized? |
| 12 | Are you involved in decisions regarding the patient treatment? |
| 13 | Do you think there's time for more education during the interviews or do you find it limited? |
| 14 | What do you ask about the medicine? What are the patient concerns regarding this topic? |
| 15 | How would you improve the outpatient visits? |
| Discharge | |
| 1 | During the discharge, after the patient had the heart attack and is ready to go home - What information do you give to them? |
| 2 | What are their main concerns? |
| 3 | How do you think it could be improved? |
| 4 | Do you use any materials such as texts, images or videos during the interview? (if so, related to what?) |
| 5 | What would be the main difference between the discharge and the rest of the interviews? |
| 6 | Do you think they are or could be connected? |
