# Supplementary material for: Perspectives of Patients and Professionals on Information and Education After Myocardial Infarction With Insight for Mixed Reality Implementation: Cross-Sectional Interview Study
Source: JMIR Hum Factors. 2020 Jun 23;7(2):e17147. doi: 10.2196/17147 (PMC7381062; doi:10.2196/17147)
Supplement: Multimedia Appendix 5 [file humanfactors_v7i2e17147_app5.docx]

**Appendix E:** Full patient journey (added as a vector file)
